# Non-Hodgkin’s lymphoma presenting as a retroperitoneal mass in the emergency department: a case report

**DOI:** 10.3389/fmed.2026.1768275

**Published:** 2026-05-13

**Authors:** Ting Liu, Yuhong Zhao, Lupeng Cui, Hanyuan Liu, Fengjiang Qu

**Affiliations:** 1 Emergency Nursing Department, The First Hospital of Jilin University, Changchun, China; 2 Department of Emergency Surgery, The First Hospital of Jilin University, Changchun, China

**Keywords:** acute dyspnea, differential diagnosis, emergency medicine, non-Hodgkin lymphoma, retroperitoneal mass

## Abstract

Non-Hodgkin’s lymphoma (NHL) is a common lymphatic system malignancy that typically presents with painless lymphadenopathy. Acute symptoms resulting from extranodal involvement are rare and may lead to diagnostic challenges in the emergency setting. We report a case of NHL in a 39-year-old male patient who presented to the emergency department with abdominal distension, oliguria, and acute dyspnea. Imaging revealed a large retroperitoneal mass with bilateral pleural effusions and ascites. The patient underwent symptomatic treatment and was admitted to the ICU. Histopathological examination of the mass confirmed the diagnosis of non-Hodgkin’s lymphoma. This case highlights the importance of considering lymphoma in the differential diagnosis of retroperitoneal masses with acute systemic manifestations and emphasizes a cautious approach to surgical intervention in the emergency setting.

## Introduction

Retroperitoneal masses are uncommon, with an incidence of 0.1–0.2%, and encompass a wide differential diagnosis, including primary sarcomas, metastatic tumors, and lymphoproliferative disorders (1, 2). Non-Hodgkin’s lymphoma (NHL) presenting primarily as a retroperitoneal mass with acute symptoms is rare and often misdiagnosed (3). Emergency physicians must consider this entity when faced with unexplained ascites, pleural effusions, and signs of lymphatic obstruction. We present a case that illustrates the diagnostic reasoning and management decisions in such a scenario.

## Case information

A 39-year-old previously healthy male patient presented to the emergency department on 18 April 2022 with a 6-day history of progressive abdominal distension and oliguria, along with acute dyspnea for 3 h. He denied fever, night sweats, weight loss, and prior similar episodes. There was no history of medication use, smoking, or alcohol consumption, nor any family history of hematologic malignancies.

**Figure 1:**
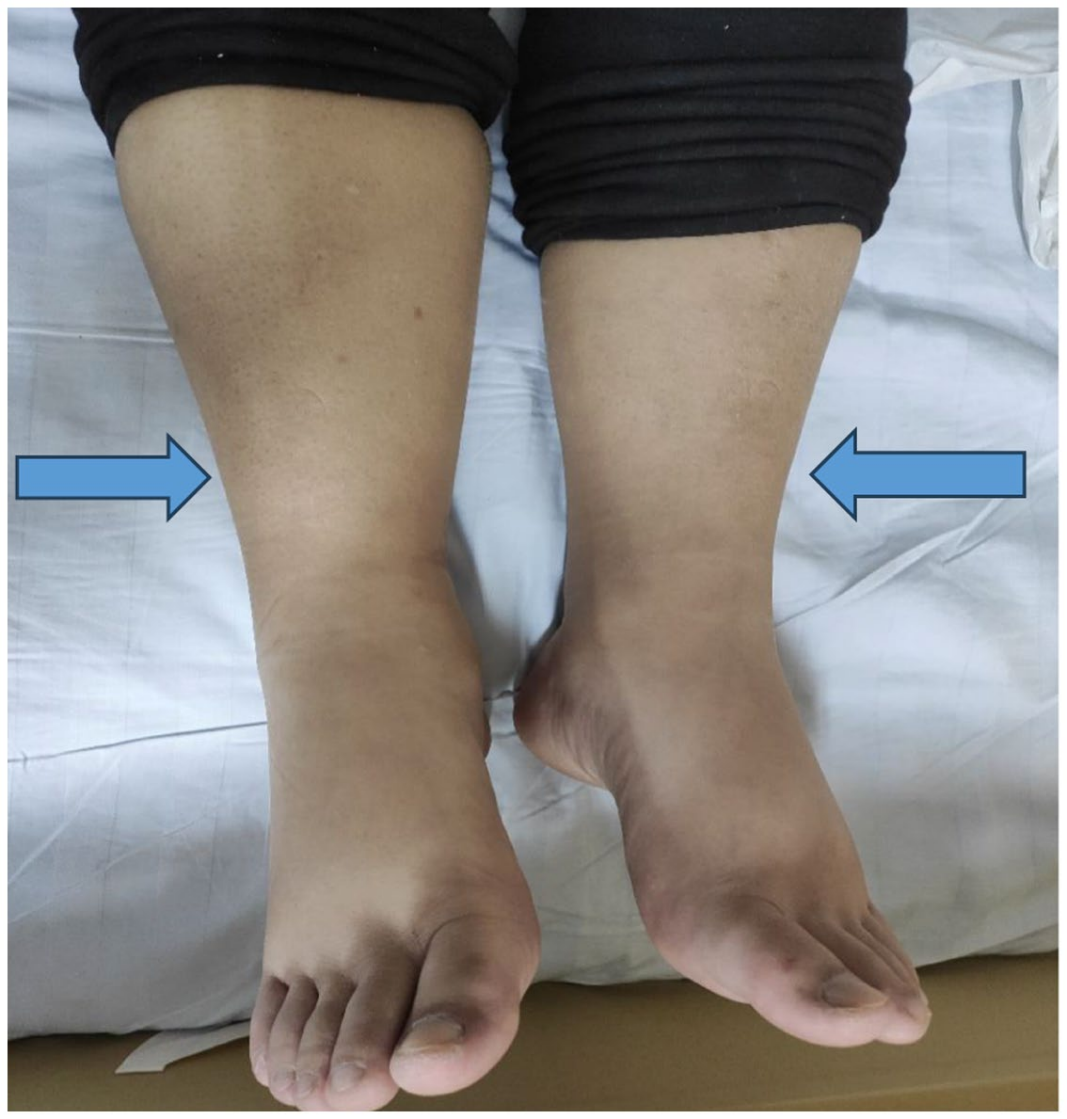
Photograph of the lower extremities showing symmetric pitting edema (arrows).

Physical examination: He appeared acutely ill, sitting upright with tachypnea. Vital signs: Blood pressure was 117/96 mmHg, heart rate 126 bpm, respiratory rate 26 bpm, and oxygen saturation 95% on room air. Lung auscultation revealed diminished breath sounds at both bases, without rales or wheezes. The abdomen was distended, with shifting dullness but no tenderness, guarding, or palpable masses. No palpable lymphadenopathy or organomegaly was noted. Symmetric pitting edema of both lower extremities was present (Figure 1). Cardiac examination showed regular tachycardia without murmurs or gallops.

Laboratory findings on admission: White blood cells: 15.11 × 10^9^/L (3.5–9.5); hemoglobin: 13.2 g/dL (13.0–17.0); platelets: 298 × 10^9^/L (125–350); urea: 10.58 mmol/L (2.9–8.2); creatinine: 146.5 μmol/L (59–104); sodium: 130.6 mmol/L (135–145); chloride: 97.4 mmol/L (98–107); CO₂ combining power: 17.4 mmol/L (22–29); glucose: 8.74 mmol/L (3.9–6.1); arterial blood gas (on room air): pH 7.37, PaCO₂ 24 mmHg, PaO₂ 73 mmHg, lactate 4.6 mmol/L (0.5–2.2), and base excess −9.5 mmol/L; BNP: 285 pg./mL (<100); D-dimer: 1.70 mg/L (<0.5); hs-CRP: 17.17 mg/L (<3); procalcitonin: 0.34 ng/mL (<0.5); coagulation profile and liver enzymes: Normal; and ECG: Sinus tachycardia.

**Figure 2:**
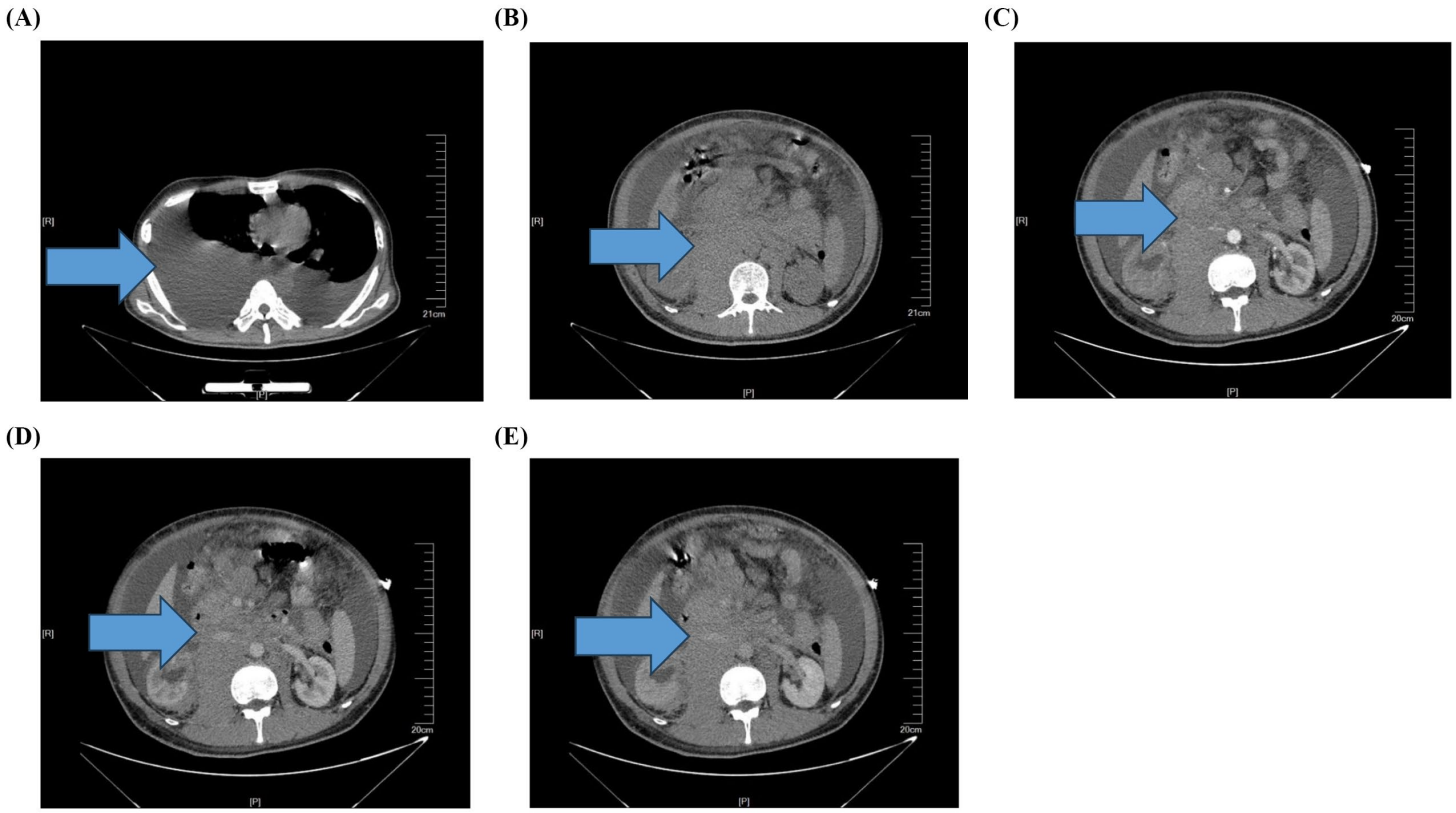
**(A)** Imaging findings: chest CT (mediastinal window) showing bilateral pleural effusions (arrows). **(B)** Imaging findings: abdominal CT plain scan: large retroperitoneal mass (arrow). **(C)** Imaging findings: arterial phase: minimal enhancement of the mass (arrow). **(D)** Imaging findings: venous phase: persistent low enhancement (arrow). **(E)** Imaging findings: delayed phase: heterogeneous enhancement (arrow).

Imaging: Chest CT (plain) showed bilateral pleural effusions with passive atelectasis (Figure 2A). Abdominal CT (plain and contrast-enhanced) revealed a large retroperitoneal mass (7.9 × 14.8 cm) with heterogeneous enhancement, poorly defined from the right adrenal gland, psoas muscle, and kidney. The mass encased the celiac trunk, superior mesenteric artery, and both renal arteries. Large ascites, mesenteric fat stranding, and extensive abdominal wall subcutaneous edema were present (Figures 2B–E). No hemorrhage was evident within the mass.

**Figure 3:**
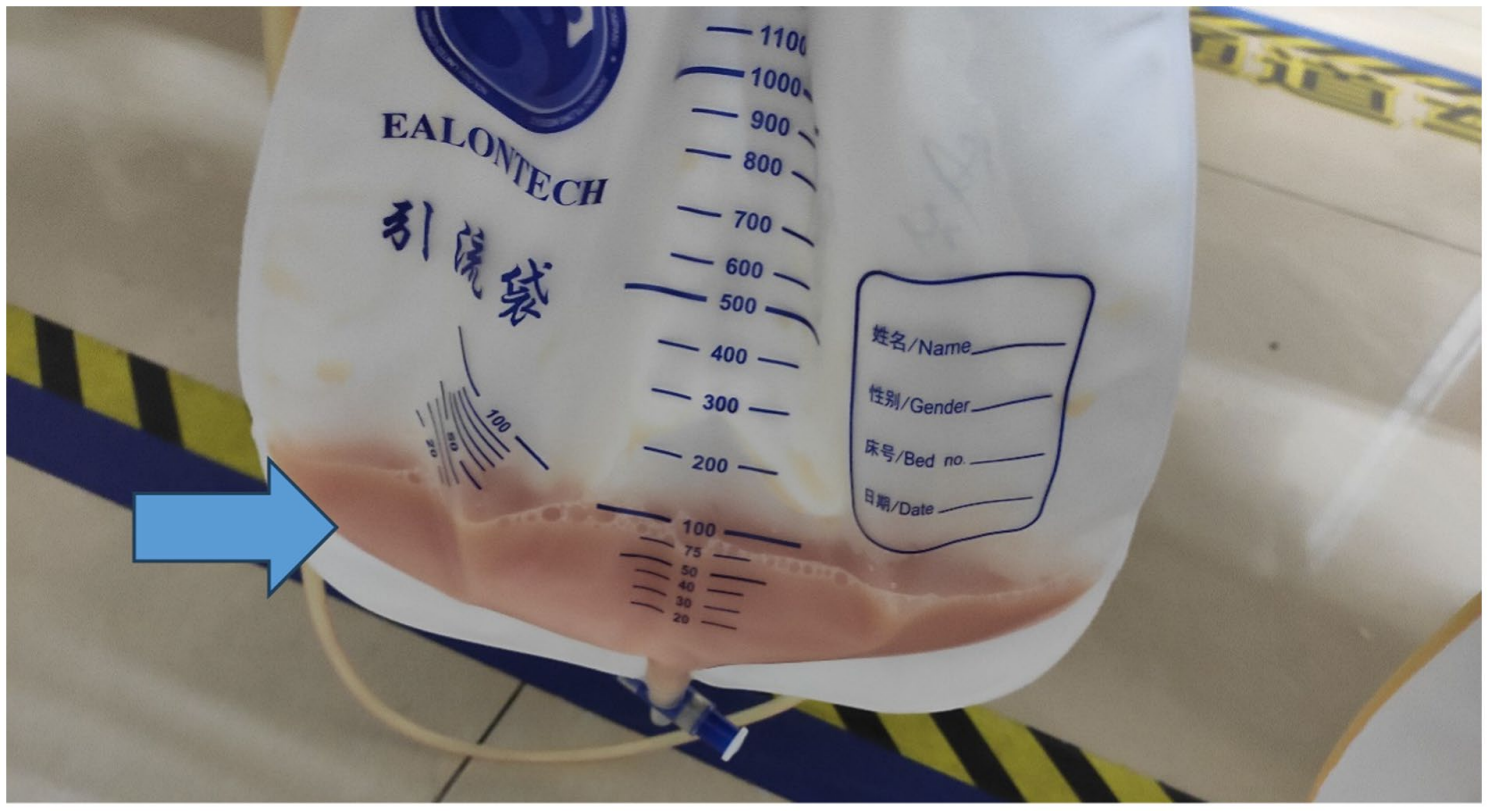
Draining yellowish pleural fluid and similar ascitic fluid.

Emergency management and initial hospital course: The patient received low-flow nasal cannula oxygen and symptomatic treatment, including diuretics and electrolyte correction. Due to the mass’s encasement of major vessels and stable hemodynamics, surgical intervention was deferred after multidisciplinary consultation with emergency surgery, interventional radiology, and hematology. Ultrasound-guided thoracentesis and paracentesis were performed on day 1, draining 500 mL of yellowish pleural fluid and 700 mL of similar ascitic fluid (Figure 3). He was admitted to the ICU for monitoring. On ICU admission, the patient’s SOFA score was 6 (respiratory 2, cardiovascular 1, renal 2, and coagulation 1). He remained hemodynamically stable without vasopressors and required only 2 L/min oxygen. Renal function improved with fluid management. On hospital day 3, a CT-guided core needle biopsy of the retroperitoneal mass was performed without complications.

Histopathology and final diagnosis: The biopsy revealed diffuse large B-cell lymphoma (DLBCL), germinal center B-cell subtype, with immunohistochemistry showing CD20+, BCL6+, MUM1−, and Ki-67 80%. Staging PET-CT showed intense FDG uptake in the retroperitoneal mass and bilateral pleural effusions (suspicious for involvement), consistent with Ann Arbor stage IV. The patient was started on R-CHOP chemotherapy (rituximab, cyclophosphamide, doxorubicin, vincristine, prednisone) on day 10. He has completed the regular cycles and shown a partial response.

## Discussion

Lymphoma is a group of malignant tumors originating from lymph nodes or other lymphoid tissues. Pathologically, it can be divided into Hodgkin lymphoma and non-Hodgkin lymphoma (4, 5). This case illustrates an uncommon presentation of non-Hodgkin’s lymphoma as a large retroperitoneal mass with acute systemic manifestations—dyspnea, abdominal distension, and oliguria—that prompted emergency evaluation. The initial differential diagnosis included common conditions such as intestinal obstruction, sepsis, or cardiorenal syndrome, but the absence of infectious markers and the presence of chylous effusions pointed toward lymphatic obstruction (6, 7). The clinical incidence of the retroperitoneal space is as low as 0.1 to 0.2%, as pathological components of retroperitoneal occupancy are diverse, including primary retroperitoneal benign and malignant tumors, metastatic tumors, and inflammatory changes (1, 2); therefore, non-Hodgkin’s lymphoma with retroperitoneal occupancy is rare and rarely reported in the literature (8).

Usually, non-Hodgkin’s lymphoma with retroperitoneal occupancy has no obvious clinical symptoms and is usually discovered incidentally during a physical examination. However, the retroperitoneal space is large, so it can grow and develop rapidly, invade the surrounding organs and tissues, and cause corresponding clinical manifestations (6, 7). One study reported that the initial symptom of non-Hodgkin’s lymphoma presenting as a retroperitoneal mass is renal colic, resulting from hydronephrosis caused by ureteral compression by the mass (9). The nonspecific nature of the clinical presentation leads to a high rate of misdiagnosis at initial evaluation (10, 11). In our patient, contrast-enhanced CT was essential for characterizing the mass and its vascular involvement, but imaging alone could not distinguish NHL from other malignancies (12). The key diagnostic clue came from fluid analysis: Elevated triglycerides and lymphocytosis confirmed chylous ascites and chylothorax, which result from obstruction of lymphatic drainage by the tumor (9). This finding narrowed the differential diagnosis to processes involving the lymphatic system, such as lymphoma or metastatic carcinoma.

From an emergency medicine perspective, this case underscores several important principles:

1. Maintain a broad differential diagnosis when symptoms do not fit a single organ system.
2. Avoid premature surgical intervention in the absence of a definitive diagnosis, especially when major vessels are involved.
3. Prioritize stabilization and supportive care while pursuing diagnostic clarification through minimally invasive procedures (e.g., thoracentesis, paracentesis, and image-guided biopsy).
4. Engage in early multidisciplinary collaboration to guide decision-making.

The patient’s elevated lactate and metabolic acidosis were attributed to hypoperfusion from intra-abdominal compartment syndrome and possible lymphatic obstruction, rather than septic shock, as he remained normotensive and did not meet qSOFA criteria. This highlights the importance of interpreting laboratory data in the full clinical context.

Therefore, as emergency physicians, we should be alert to the possibility that the patient’s condition may be due to a systemic disease. Surgical intervention should not be performed blindly at this time; instead, stabilization of vital signs and symptomatic supportive care should be provided to alleviate the patient’s current symptoms. The appearance of the thoraco-abdominal puncture fluid suggested the possibility of lymphatic fluid. In combination with the patient’s retroperitoneal mass and involvement of major abdominal vessels, a hematologic malignancy was highly suspected. Definitive diagnosis of NHL requires histologic and immunophenotypic confirmation (5). In our case, core needle biopsy provided adequate tissue for subtyping and treatment planning. R-CHOP remains the standard for DLBCL (4). Staging with PET-CT is essential for prognosis assessment and therapy.

This report has limitations, as it describes a single case and some details (e.g., long-term follow-up) are not yet available. Nevertheless, it offers valuable lessons for emergency physicians encountering similar presentations.

## Conclusion

Non-Hodgkin’s lymphoma should be considered in the differential diagnosis of retroperitoneal masses presenting with acute symptoms. The presence of chylous effusions serves as a key diagnostic clue. A stepwise approach—comprising initial stabilization, fluid analysis, multidisciplinary consultation, and image-guided biopsy—leads to timely diagnosis and appropriate oncology referral.

## Data Availability

The datasets presented in this study can be found in online repositories. The names of the repository/repositories and accession number(s) can be found in the article/supplementary material.
